# Supplementary material for: Stepwise oxygenation of the Paleozoic atmosphere
Source: Nat Commun. 2018 Oct 4;9:4081. doi: 10.1038/s41467-018-06383-y (PMC6172248; doi:10.1038/s41467-018-06383-y)
Supplement: Supplementary file 1 — Supplementary Information [file 41467_2018_6383_MOESM1_ESM.pdf]

Stepwise oxygenation of the Paleozoic atmosphere

Supplementary information

Krause et al.

Supplementary Note 1: Model validation and further sensitivity analyses

In order to check the robustness of our new model, GEOCARBSULFOR, we conducted a number of tests, to compare various model outputs to available geologic data. We also completed a further sensitivity analysis, to measure the effect different formulations of pyrite weathering have on atmospheric oxygen levels.

We generated a synthetic ocean $\delta^{34}S$ sulphate record from the model, for comparison against the geological record (Supplementary Fig. 1). Using a static sulphur isotope fractionation factor (α_S) for the entire time period, we conducted three model runs, with: α_S = 16.7‰ – the lowest value recorded by Wu *et al.*[1]; α_S = 48.1‰ – the highest value recorded by Wu *et al.*[1]; and α_S = 35.95‰ – the average for the period 570 Ma to Present, calculated using the Wu *et al.*[1] dataset.

Our GEOCARBSULFOR model is sensitive to the fractionation factor used, but the average value generates an ocean $\delta^{34}S$ sulphate record which is in good agreement with the geologic record for almost the entire run. The highest fractionation factor provides a $\delta^{34}S$ sulphate record that closely matches the geologic record from the end-Permian onwards, and this is coincident with an actual rise to higher fractionation values[1] during this time period. Although the $\delta^{34}S$ output using the lowest fractionation value is very low for the entire run, the actual sulphur isotope fractionation is only very low (≤ 22.1‰) for the period 570 – 540 Ma, with higher values (≥ 27.5‰) for the Phanerozoic itself[1]. Model runs with separate fractionation values for the periods 570 – 540 Ma, and 530 Ma – Present, give $\delta^{34}S$ records which are closer to the geological one. Additionally, the $\delta^{34}S$ output is sensitive to the starting $\delta^{34}S$ value of the sulphur reservoir. We use a value of 23‰ for 570 Ma, following Wu *et al.*[1]

for consistency, however, using the original GEOCARBSULF value of 35.2‰ and the highest fractionation (48.1‰) produces a $\delta^{34}S$ record which for the last 250 Ma is near identical to the geologic record.

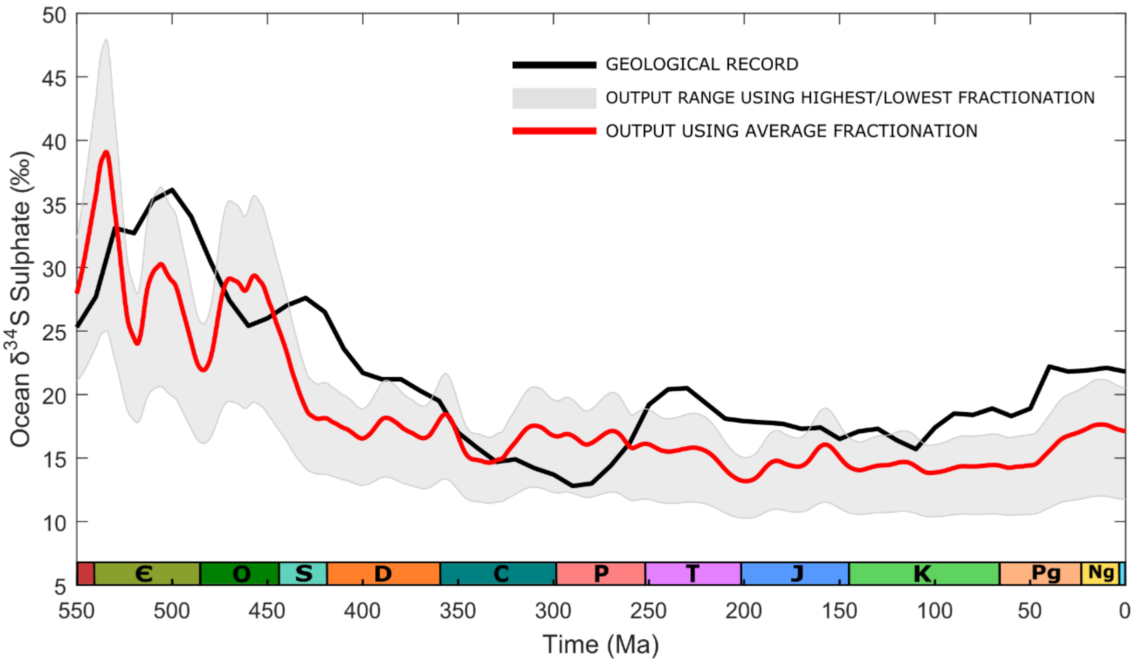

Supplementary Figure 1 | Ocean sulphate isotope predictions for the Phanerozoic from our GEOCARBSULFOR model. The red line is the ocean $\delta^{34}S$ output using a static average isotopic fractionation from 570 Ma to Present (35.95‰), calculated from the Wu et al.[1] dataset. The grey envelope is the ocean $\delta^{34}S$ output using the lowest (16.7‰) and highest (48.1‰) fractionation values from Wu et al.[1] The black line is the geologic $\delta^{34}S$ data from Wu et al. [1]

We conducted a test to ascertain whether simply substituting the sulphur isotope fractionation equation in the original GEOCARBSULF with the Wu *et al.*[1] fractionation data, would produce low pO_2 in the early Paleozoic, as Wu *et al.*[1] take into account the effects of

reoxidation and disproportionation on sulphide (though not the possibility of other sinks, such as organic sulphur) changing the isotopic signature of sedimentary pyrite. This change leads to unfeasibly high (considering geochemical redox data[e.g.2,3]) oxygen concentrations of >35% atm in the early Paleozoic, and negative O_2 during the late-Mesozoic to Cenozoic. Therefore, a pO_2 dependent feedback embedded somewhere in the sulphur cycle is a requirement for the model to produce reasonable levels of atmospheric O_2.

As GEOCARBSULFOR can calculate the amount of pyrite and sulphate being buried at each time-step, we examined whether the model was generating reasonable values for the fraction of total sulphur leaving the ocean as pyrite (f_{pyr}), by comparing the model outputs to other modelling approaches[4,5]. We tried two different methods to compute f_{pyr}: one was a direct method, using the burial fluxes, following supplementary equation (57). The second method uses our synthetic ocean δ^{34}S sulphate record and static α_s values from Supplementary Fig. 1 to calculate records for the δ^{34}S sedimentary pyrite and sulphate, which, combined with a constant $\delta^{34}S_{in}$ value of 5‰[4], can be used to calculate f_{pyr} using supplementary equation (58).

The direct method generates f_{pyr} values (Supplementary Fig. 2 [A]) which are similar to the various parameterized models by Halevy *et al.*[5], but are not as high as those predicted by Canfield and Farquhar[4] in the early Paleozoic. However, GEOCARBSULFOR does end with an f_{pyr} value for the present day which is in line with all the different models, and there is a noticeable transition from higher f_{pyr} values in the early Paleozoic, to lower values for the rest of the Phanerozoic. Our isotope method (Supplementary Fig. 2 [B]) generates f_{pyr} values which also decrease over the course of the Phanerozoic. Using a static α_s value of 16.7‰ delivers f_{pyr} values that are equal or close to those predicted by Canfield and Farquhar[9] for the

early Paleozoic and for much of the Mesozoic and Cenozoic, but are too high for the Permian and Triassic. The static α_s value of 48.1‰ predicts comparable results to Canfield and Farquhar[9] for the Permian, Triassic and modern-day, but are otherwise too low. Both static values result in f_{pyr} values which compare favourably with Halevy *et al.*[5]. As with our synthetic ocean $\delta^{34}S$ sulphate record in Supplementary Fig. 1, these f_{pyr} results suggest a transition from lower to higher (persistently >35.9‰ – the average value over the last 570 Ma) sulphur isotope fractionation values in the Permian, and this can be seen in the geologic record.

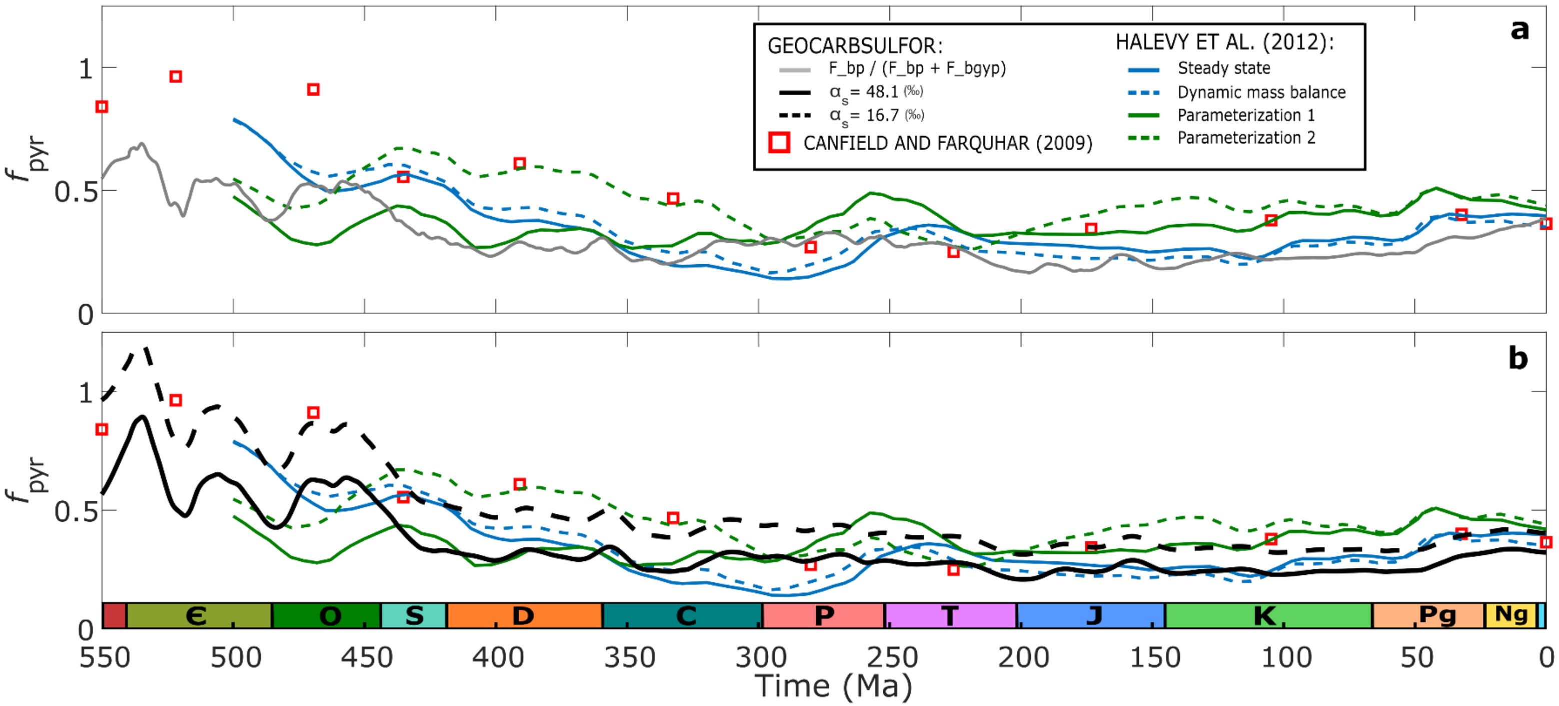

Supplementary figure 2 | The fraction of total sulphur leaving the ocean as pyrite in GEOCARBSULFOR. [A] The grey line is the fraction of total sulphur leaving the ocean as pyrite, calculated by GEOCARBSULFOR's burial fluxes over time. [B] The black lines are the fraction of total sulphur leaving the ocean as pyrite, calculated by GEOCARBSULFOR using the synthetic ocean $\delta^{34}S$ sulphate record and supplementary equation (58); the solid black line uses a static α_s of 48.1‰ and the dashed black line uses a static α_s of 16.7‰. The red squares in [A] and [B] are f_{pyr} values taken from Canfield and Farquhar[4], the blue and green lines are taken from different input model runs in Halevy et al.[5]

In GEOCARBSULF[6], Berner contends that erosive stripping is a more important process in the weathering of pyrite (and organic matter) than oxygen levels, and although the recent iteration of COPSE[7] concurs, the original COPSE[8] model and others[9,10], include a dependency on atmospheric oxygen levels when considering the weathering of reductants (e.g. pyrite). In Supplementary Fig. 3 we conduct a sensitivity analysis of the model to an oxidative feedback term in the equations for young and ancient pyrite weathering, as well as an analysis of the importance of linking young pyrite weathering to the weathering of silicates.

In Supplementary Fig. 3, the black line is our GEOCARBSULFOR baseline, while the grey line is almost the same, but with the oxidative feedback ($O_2mr^{0.5}$) removed from the pyrite weathering equations (equation (3) and supplementary equation (17)). The magenta line is a model identical to GEOCARBSULFOR but the pyrite weathering equations are those used in the original GEOCARBSULF[6], while the green line is the same as the magenta line, but with oxidative feedback included into Berner's equations. Finally, the yellow line is as the magenta line, but with a fixed uplift rate of 1, for the entire run.

Our results show that although there is some sensitivity to the inclusion of an oxidative feedback term in pyrite weathering, it does not alter the general trend of the evolution of pO_2 as predicted by GEOCARBSULFOR. Comparison of our baseline GEOCARBSULFOR (black line) to no oxidative feedback (grey line) reveals a difference of ~1 – 2% atm in predicted oxygen levels for most of the model run, apart from the Devonian which has a difference of ~2 – 3% atm, both of which would likely lie within an error analysis of the

model. Similarly, tying the weathering of pyrite to the weathering of silicates makes little difference to oxygen predictions for much of the Phanerozoic, with the only real difference in predictions (green line compared to black) coming in the Cretaceous, where the effect of uplift (which is included in the silicate weathering equation – supplementary equation (40)) is dampened in GEOCARBSULFOR.

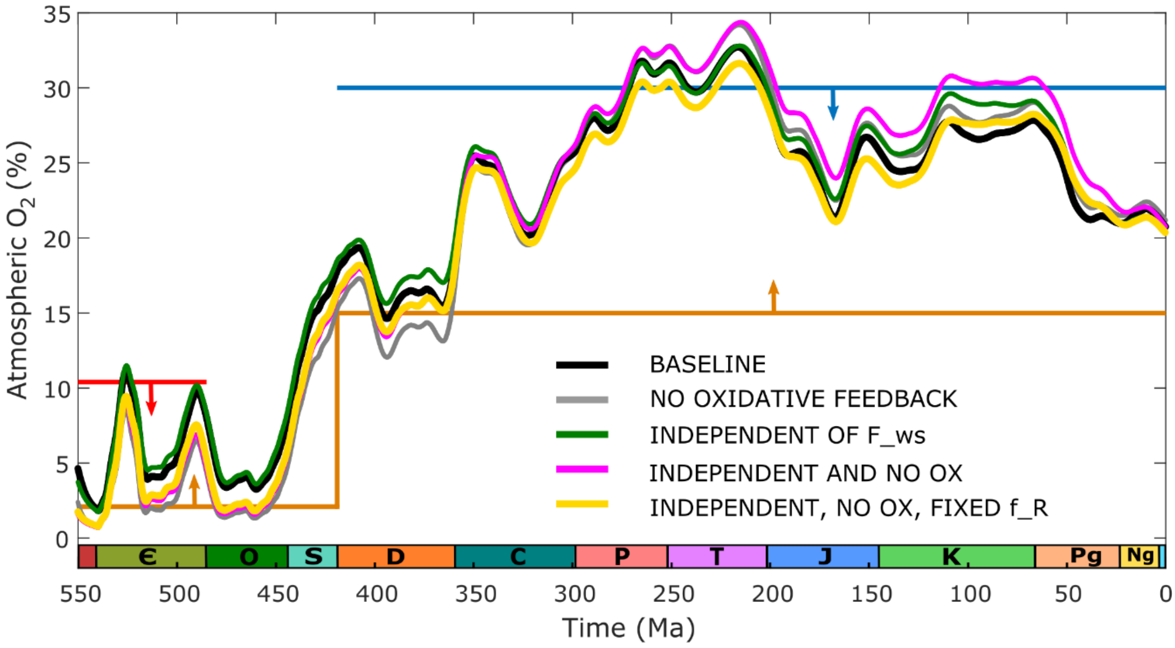

Supplementary figure 3 | Pyrite weathering sensitivity tests. The black line is the baseline GEOCARBSULFOR oxygen (as seen in Fig. 3 in the main text). The grey line includes no oxidative feedback in the pyrite weathering equations, while the green line decouples pyrite weathering from silicate weathering. The magenta line has no oxidative feedback and is not linked to silicate weathering. The yellow line is the same as the magenta line but uplift is fixed (to the modern-day value of 1) for the entire Phanerozoic.

Two final tests were conducted, to assess the robustness of our model outputs. These tests were to evaluate both the amount of sulphate assigned to the ocean-atmosphere reservoir by the model (Supplementary Fig. 4), and the CO_2 levels generated (Supplementary Fig. 5), against other models and geochemical proxies. In both tests, GEOCARBSULFOR outputs compare favourably with both the proxy data and other modelling efforts. CO_2 levels predicted by GEOCARBSULFOR are on the low side of the proxy bands, particularly during the Cretaceous and Paleogene. The Paleogene low is a common issue with previous work involving GEOCARBSULF[11–13], and requires the seafloor spreading rate to be ~3x the present day value in order to reconcile the model predictions with the proxy record. The discrepancy between model predictions and proxy estimates is possibly due to underrepresentation, or absence, of all plate boundary types and their contribution to CO_2 degassing[14]. However, recent work on this issue has resulted in GEOCARBSULF predictions that better match the Paleogene record, but are subsequently too high for the Neogene[14]. Nevertheless, we believe that our model has been comprehensively checked against the available geologic data and provides a good fit to our understanding of various geochemical indices, and thus GEOCARBSULFOR gives a compelling indication of low pO_2 in the early Paleozoic.

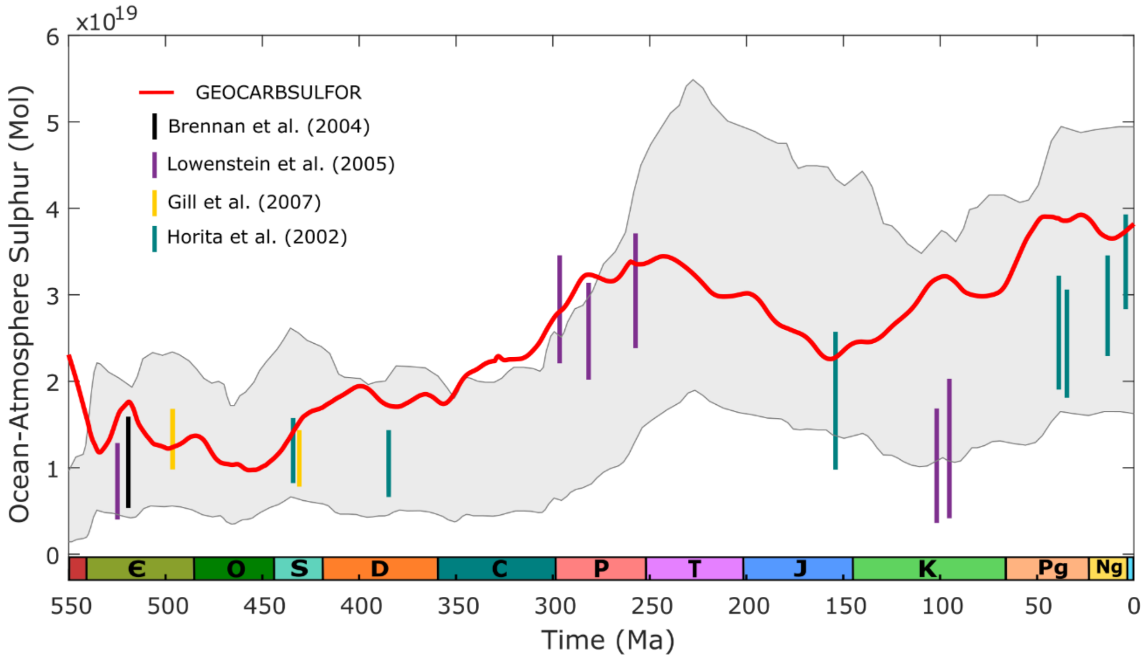

Supplementary figure 4 | The ocean-atmosphere sulphur reservoir generated by our GEOCARBSULFOR model, compared with proxies and other model outputs. The red line is the amount of sulphate in the ocean-atmosphere reservoir throughout the Phanerozoic as generated by our GEOCARBSULFOR model. The vertical lines are estimates based on fluid-inclusion (Horita et al.[15]; Brennan et al.[16]; Lowenstein et al.[17]) and modelling studies (Gill et al.[18]). The grey envelope is the ± 1σ uncertainty range from Algeo et al.[19]

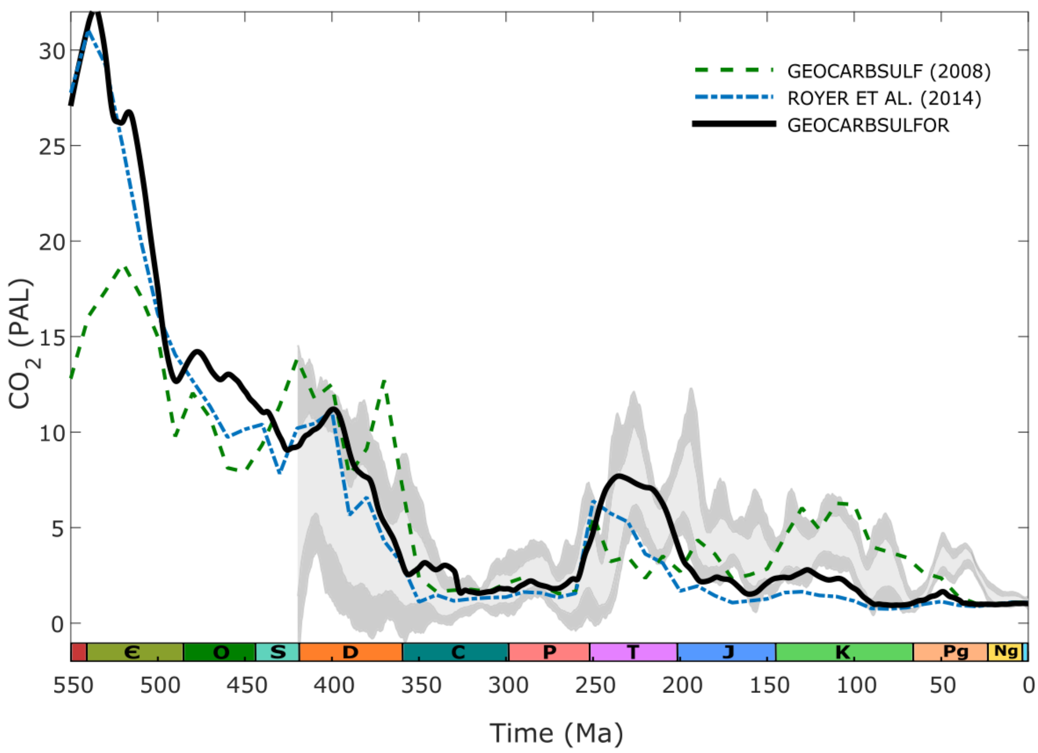

Supplementary figure 5 | Atmospheric CO_2 levels generated by our GEOCARBSULFOR model, compared with other model outputs and CO_2 proxies. The black line is our GEOCARBSULFOR model output, the green dashed line is taken from Berner[11] where basalt/granite weatherability = 2, *NV* = 0.015, fB(0) = 5, the blue dot-dash line is the median output from Royer et al.[12] and the light and dark grey bands are 68% and 95% confidence intervals, respectively, of CO_2 proxy data[20]. Outputs are measured as multiplications of the present atmospheric level (PAL), which in GEOCARBSULF is taken as the mean Quaternary value of 250ppm.

Finally, to ensure completeness, we checked that the model adequately apportions the total mass of sedimentary sulphur between the gypsum and pyrite reservoirs, over time, starting with a pyrite-dominated Paleozoic[4], and finishing with near equal reservoir values (total pyrite of 180 x 10^{18} moles and total gypsum of 200 x 10^{18} moles) in the Present[21];

GEOCARBSULFOR achieves this (Supplementary Fig. 6), with values at the end of its run that are very close to those at Present, indicating that our model is robust.

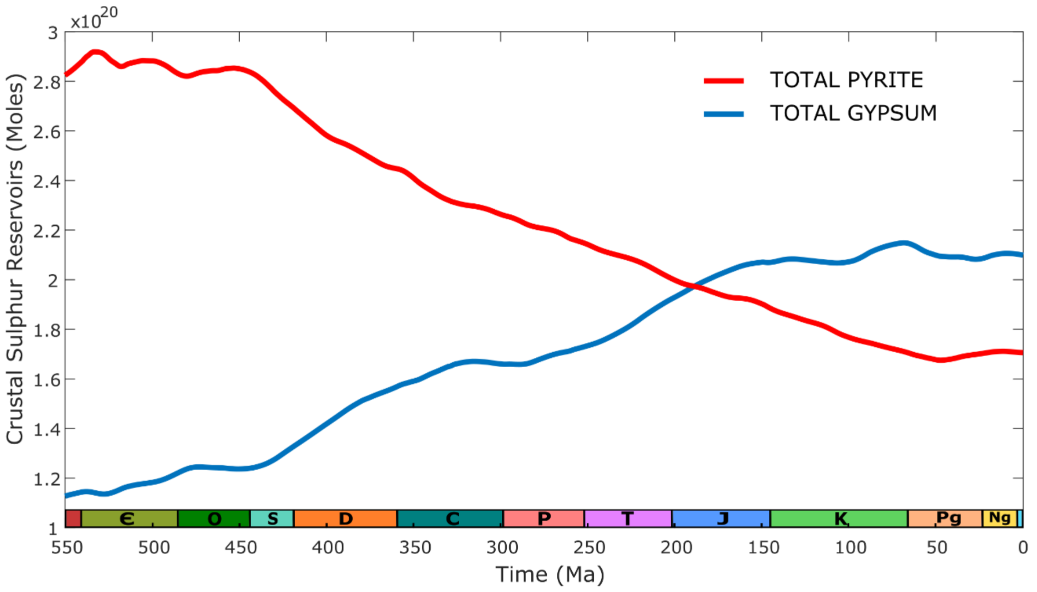

Supplementary figure 6 | GEOCARBSULFOR predictions of total sedimentary sulphide (pyrite) and total sedimentary sulphate (gypsum) over the Phanerozoic. The red line is total pyrite (young plus ancient reservoirs), and the blue line is total gypsum (young plus ancient reservoirs).

Supplementary Note 2: Full model parameters

For the following tables, where we mention GEOCARBSULF as a source, we mean the final values as used in the 2009[22] paper, some of which are unchanged from, for example, GEOCARB II[23].

Supplementary table 1: Present day parameters used in GEOCARBSULFOR

Parameter	Algebraic Representation	Value	Source
Atmosphere and ocean CO_2	A0	3.193×10^{18} mol	COPSE[8]
Atmosphere and ocean O_2	O0	3.8×10^{19} mol	GEOCARBSULF[22]
Atmosphere and ocean sulphate	OA_S_0	3.8×10^{19} mol	GEOCARBSULF
Young buried pyrite sulphur*	*Pyr*_0	12.8571×10^{18} mol	COPSE / GEOCARBSULF
Young buried gypsum sulphur*	*Gyp*_0	100×10^{18} mol	COPSE / GEOCARBSULF
Temperature	*temp*_0	288 K	
Silicate weathering	*F*_ws_0	6.67×10^{18} mol C myr^{-1}	GEOCARBSULF
Young carbonate weathering*	*F*_wc_y0	11.35×10^{18} mol C myr^{-1}	COPSE / GEOCARBSULF
Burial rate of organic carbon	*F*_bg_0	5×10^{18} mol C myr^{-1}	Berner and Canfield[24]
Rate of exchange of Ca and Mg between basalt and seawater	*F*_bo_0	5×10^{18} mol myr^{-1}	GEOCARBSULF
Fraction of total Ca and Mg	Xvolc_0	0.35	GEOCARBSULF

silicate weathering derived from volcanic rocks			

* Values are taken from COPSE but then split into young and ancient values following the proportions set out in GEOCARBSULF. Only present young values are required for the model.

Supplementary table 2: Constant parameters used in GEOCARBSULFOR

Parameter	Algebraic Representation	Value	Source
Normalized activation energy for silicates	*ACT*_si	0.09 K^{-1}	GEOCARBSULF
Normalized activation energy for carbonates	*ACT*_carb	0.087 K^{-1}	GEOCARBSULF
Effect of solar insolation on temperature	Ws	7.4°	GEOCARBSULF
Proportion of plants fertilized by increasing CO_2	*FERT*	0.4	GEOCARBSULF
Curve fitting parameter for effect of O_2 on $\delta^{13}C$ fractionation	*J*	4	GEOCARBSULF
Average value of $^{87}Sr/^{86}Sr$ of sub-aerial and submarine volcanic rocks	*Rv*	0.704	GEOCARBSULF
Ratio of chemical weathering in volcanic to non-volcanic silicate rocks	*VNV*	2	GEOCARBSULF
Arbitrary parameter used to calculate the average value for	*NV*	0.015	GEOCARBSULF

$^{87}Sr/^{86}Sr$ of non-volcanic silicate rocks			
Young pyrite weathering rate constant*	*new_kwp*	$4.6x10^{17}$ my^{-1}	For steady state
Young gypsum weathering rate constant*	*new_kwgyp*	$1.2x10^{18}$ my^{-1}	For steady state
Young organic carbon weathering rate constant	*k*_wg	0.018 my^{-1}	GEOCARBSULF
Young carbonate weathering rate constant	*k*_wc	0.018 my^{-1}	GEOCARBSULF
Ancient pyrite weathering rate constant	*kwpa*	0.000893 my^{-1}	For steady state
Ancient gypsum weathering rate constant	*kwgypa*	0.0033 my^{-1}	For steady state
Ancient organic carbon weathering rate constant	*kwga*	0.0005 my^{-1}	For steady state
Ancient carbonate weathering rate constant	*kwca*	0.00125 my^{-1}	For steady state
Ancient pyrite degassing rate constant	*kmpa*	0.000893 my^{-1}	For steady state
Ancient gypsum degassing rate constant	*kmgypa*	0.0033 my^{-1}	For steady state
Ancient organic carbon degassing rate constant	*kmga*	0.0005 my^{-1}	For steady state
Ancient carbonate degassing rate constant	*kmca*	0.001668 my^{-1}	For steady state
Pyrite burial rate constant*	*k*_bp	$1.15x10^{18}$ my^{-1}	For steady state
Gypsum burial rate constant*	*k*_bgyp	$1.6x10^{18}$ my^{-1}	For steady state
Pyrite young to ancient flux rate constant	*k*_p_ya	0.01 my^{-1}	For steady state
Gypsum young to ancient flux rate constant	*k*_gyp_ya	0.01 my^{-1}	For steady state

Organic carbon young to ancient flux rate constant	*k*_g_ya	0.018 my^{-1}	For steady state
Carbonate young to ancient flux rate constant	*k*_c_ya	0.018 my^{-1}	For steady state

* The values for these parameters are so much larger as they are multiplied by normalized reservoirs, instead of a reservoir at time (t).

Supplementary table 3: Time dependent parameters used in GEOCARBSULFOR

Parameter	Algebraic Representation	Source	Notes
Total land area	*f*_A	Royer *et al.*[12]	Time array
Fraction of land area experiencing chemical weathering	*f*_AW	Royer *et al.*	Time array
Global river runoff	*f*_D	Royer *et al.*	Time array
Effect of change in paleogeography on temperature	*GEOG*	Royer *et al.*	Time array
Proportion of land area underlain by carbonates	*f*_L	Royer *et al.*	Time array
Degassing rate due to tectonics / seafloor spreading rate*	*f*_G or *f*_SR	GEOCARBSULF	Time array - the same values for this parameter are used by Royer *et al.*
Ocean $\delta^{13}C$ carbonate record	*delta*_OA_C	Saltzman and Thomas[25]	Time array
Normalized calcium reservoir	*Calc*	Horita *et al.* (2002)[15]	Time array – generated from Ca fluid inclusion data
Dependence of weathering rate on soil	*f*_E	GEOCARBSULF	0-80 Ma *f*_E = 1<br>130-350 Ma *f*_E = 0.875

biological activity due to land plants			380-550 Ma *f*_E = 0.25 Linear interpolation between 80-130 Ma and 350-380 Ma
Mean land elevation	*f*_R	GEOCARBSULF	Cubic fit – See supplementary equations (2) and (3)
Dependence of degassing rate on relative proportions of carbonates on shallow platforms or deep ocean	*f*_C	GEOCARBSULF	150-550 Ma *f*_C = 0.75 0-150 Ma is a time dependent equation, see supplementary equation (1)
Ocean ^{87}Sr record for use in calculating silicate weathering	*R*_ocm	Royer *et al.*	Time array
Response of global mean temperature to atmospheric CO_2 greenhouse effect	*gamma*	Royer *et al.*	For 0-40 Ma and 260-330 Ma: *gamma* = 8.6562<br>All other time: *gamma* = 4.3281
Temperature controlled runoff factor	*RUN*	GEOCARBSULF	0-40 Ma and 260-340 Ma: *RUN* = 0.045<br>All other time: *RUN* = 0.025
Effect of changes to CO_2 on carbonate and silicate weathering	*f*_CO2	GEOCARBSULF	0-350 Ma: f_CO2 = *VAS*<br>380-550 Ma: f_CO2 = *NOVAS*<br>Linear interpolation between 350-380 Ma - see supplementary equations (7 – 12)

* Both of these terms are used throughout the literature, but in GEOCARBSULF they are treated as the same thing.

For all time arrays we apply interpolation between data points.

Supplementary table 4: Starting reservoir sizes of GEOCARBSULFOR

Parameter	Algebraic Representation	Value	Source
Ocean and atmosphere sulphur	OA_S	$38\text{x}10^{18}$ mol	GEOCARBSULF
Ocean and atmosphere carbon	OA_C	$35.5\text{x}10^{18}$ mol	For attaining PAL
Young pyrite	*Pyr*_y	$20\text{x}10^{18}$ mol	This study
Ancient pyrite	*Pyr*_a	$260\text{x}10^{18}$ mol	This study
Young gypsum	*Gyp*_y	$50\text{x}10^{18}$ mol	This study
Ancient gypsum	*Gyp*_a	$50\text{x}10^{18}$ mol	This study
Young organic carbon	*G*_y	$250\text{x}10^{18}$ mol	GEOCARBSULF
Ancient organic carbon	*G*_a	$1000\text{x}10^{18}$ mol	GEOCARBSULF
Young carbonate	*C*_y	$1000\text{x}10^{18}$ mol	GEOCARBSULF
Ancient carbonate	*C*_a	$4000\text{x}10^{18}$ mol	GEOCARBSULF
Young organic carbon isotope mass balance	*delta*_g_y · *G*_y	$-23.5 \cdot 250\text{x}10^{18}$	GEOCARBSULF
Ancient organic carbon isotope mass balance	*delta*_g_a · *G*_a	$-23.5 \cdot 1000\text{x}10^{18}$	GEOCARBSULF
Young carbonate isotope mass balance	*delta*_c_y · *C*_y	$3 \cdot 1000\text{x}10^{18}$	GEOCARBSULF
Ancient carbonate isotope mass balance	*delta*_c_a · *C*_a	$-1.5774 \cdot 4000\text{x}10^{18}$	GEOCARBSULF

Supplementary Note 3: Full model equations

The full set of equations for the model are documented below, except the new burial equations for pyrite and gypsum, and weathering equations for young pyrite and young gypsum (equations 3 – 6), which can be found in the Methods section of the main paper. These are taken from the COPSE[8] model. All other equations have been taken from GEOCARBSULF, but we note where alterations to the original equations have been made.

Supplementary table 5: Flux and parameter equations

f_C for t < 150 Ma:	$f\_C = 0.75 + \left[\left(\frac{0.25}{150}\right) \cdot (150 + t)\right]$	[1]
Mean land elevation:	$f\_R = \left(\frac{f\_RT}{1.063}\right)^{0.67}$	[2]
Where:	$f\_RT = \left(25.269 \cdot \left(\frac{t}{1000}\right)^3\right) + \left(26.561 \cdot \left(\frac{t}{1000}\right)^2\right) + \left(6.894 \cdot \left(\frac{t}{1000}\right)\right) + 1.063$	[3]
Ratio of CO_2 relative to present day:	$RCO_2 = \frac{CO_2(t)}{A0}$	[4]
Ratio of O_2 relative to present day:	$O_2mr = \frac{O_2(t)}{O0}$	[5]
Conversion of relative O_2 to atmospheric %:	$O_2atm = \left(\frac{O_2mr}{O_2mr + 3.762}\right) \cdot 100$	[6]

Effect of changes in CO_2 on carbonate and silicate weathering:	$VAS = \left(\frac{2 \cdot \mathrm{RCO2}}{1 + \mathrm{RCO2}}\right)^{FERT}$ $NOVAS = \mathrm{RCO2}^{0.5}$	[7]
Global average temperature:	$temp = (gamma - \ln \mathrm{RCO2}) - \left(\mathrm{Ws} \cdot \frac{t}{-570}\right) + GEOG + temp\_0$	[8]
Effect of changes in temperature on silicate weathering:	$f\_\mathrm{Tsi} = \mathrm{e}^{(ACT\_\mathrm{si} \cdot [temp - temp\_0])} \cdot \{1 + [RUN \cdot (temp - temp\_0)]\}^{0.65}$	[9]
Combined effect of changes to temperature and CO_2 on silicate weathering:	$\mathrm{fBsi} = f\_\mathrm{Tsi} \cdot f\_\mathrm{CO2}$	[10]
Effect of changes in temperature on carbonate weathering:	$f\_\mathrm{Tcarb} = 1 + [ACT\_\mathrm{carb} \cdot (temp - temp\_0)]$	[11]
Combined effect of changes to temperature and CO_2 on carbonate weathering:	$f\_\mathrm{BBcarb} = f\_\mathrm{Tcarb} \cdot f\_\mathrm{CO2}$	[12]
*Weathering of young organic carbon:	$F\_\mathrm{wg\_y} = f\_\mathrm{R} \cdot f\_\mathrm{A} \cdot k\_\mathrm{wg} \cdot G\_\mathrm{y}(t) \cdot \mathrm{O_2mr}^{0.5}$	[13]
*Weathering of ancient organic carbon:	$F\_\mathrm{wg\_a} = f\_\mathrm{R} \cdot kwga \cdot G\_\mathrm{a}(t) \cdot \mathrm{O_2mr}^{0.5}$	[14]

Weathering of young carbonate:	$F\_\text{wc\_y} = f\_\text{BBcarb} \cdot f\_\text{L} \cdot f\_\text{A} \cdot f\_\text{D} \cdot f\_\text{E} \cdot \ k\_\text{wc} \cdot \ C\_\text{y}(t)$	[15]
Weathering of ancient carbonate:	$F\_\text{wc\_a} = f\_\text{BBcarb} \cdot f\_\text{L} \cdot f\_\text{A} \cdot f\_\text{D} \cdot f\_\text{E} \cdot \ kwca \cdot \ C\_\text{a}(t)$	[16]
*Weathering of ancient pyrite:	$F\_\text{wp\_a} = f\_\text{R} \cdot kwpa \cdot \ Pyr\_\text{a}(t) \cdot \ \text{O}_2\text{mr}^{0.5}$	[17]
*Weathering of ancient gypsum:	$F\_\text{wgyp\_a} = f\_\text{A} \cdot f\_\text{D} \cdot kwgypa \cdot \ Gyp\_\text{a}(t)$	[18]
*Metamorphism / degassing of ancient pyrite:	$\text{Fmp} = f\_\text{SR} \cdot kmpa \cdot \ Pyr\_\text{a}(t)$	[19]
*Metamorphism / degassing of ancient gypsum:	$\text{Fms} = f\_\text{SR} \cdot kmgypa \cdot \ Gyp\_\text{a}(t)$	[20]
*Metamorphism / degassing of ancient organic carbon:	$\text{Fmg} = f\_\text{SR} \cdot kmga \cdot \ G\_\text{a}(t)$	[21]
*Metamorphism / degassing of ancient carbonate:	$\text{Fmc} = f\_\text{SR} \cdot f\_\text{C} \cdot kmca \cdot \ C\_\text{a}(t)$	[22]
Fractionation of δ^{13}C:	$alpha\_\text{C} = 27 + \ [J \cdot \ (\text{O}_2\text{mr} - 1)]$	[23]
Young organic carbon isotopes:	$delta\_\text{g\_y} = \dfrac{delta\_\text{g\_y}(t) \ \cdot \ G\_\text{y}(t)}{G\_\text{y}(t)}$	[24]
Ancient organic carbon isotopes:	$delta\_\text{g\_a} = \dfrac{delta\_\text{g\_a}(t) \ \cdot \ G\_\text{a}(t)}{G\_\text{a}(t)}$	[25]

Young carbonate isotopes:	$$delta\_\mathrm{c\_y} = \frac{delta\_\mathrm{c\_y}(t) \cdot C\_\mathrm{y}(t)}{C\_\mathrm{y}(t)}$$	[26]
Ancient carbonate isotopes:	$$delta\_\mathrm{c\_a} = \frac{delta\_\mathrm{c\_a}(t) \cdot C\_\mathrm{a}(t)}{C\_\mathrm{a}(t)}$$	[27]
Burial of organic carbon:	$$F\_\mathrm{bg} = \frac{1}{alpha\_\mathrm{C}} \cdot \{[(delta\_\mathrm{OA\_C} - delta\_\mathrm{C\_y}) \cdot F\_\mathrm{wc\_y}] + [(delta\_\mathrm{OA\_C} - delta\_\mathrm{C\_a}) \cdot F\_\mathrm{wc\_a}] + [(delta\_\mathrm{OA\_C} - delta\_\mathrm{G\_y}) \cdot F\_\mathrm{wg\_y}] + [(delta\_\mathrm{OA\_C} - delta\_\mathrm{G\_a}) \cdot F\_\mathrm{wg\_a}] + [(delta\_\mathrm{OA\_C} - delta\_\mathrm{C\_a}) \cdot \mathrm{Fmc}] + [(delta\_\mathrm{OA\_C} - delta\_\mathrm{G\_a}) \cdot \mathrm{Fmg}]\}$$	[28]
*Burial of carbonate:	$$F\_\mathrm{bc} = F\_\mathrm{ws} + F\_\mathrm{wc\_y} + F\_\mathrm{wc\_a}$$	[29]
*Young to ancient pyrite flux:	$$F\_\mathrm{ya\_p} = k\_\mathrm{p\_ya} \cdot Pyr\_\mathrm{y}(t)$$	[30]
*Young to ancient gypsum flux:	$$F\_\mathrm{ya\_gyp} = k\_\mathrm{gyp\_ya} \cdot Gyp\_\mathrm{y}(t)$$	[31]
*Young to ancient organic carbon flux:	$$F\_\mathrm{ya\_g} = k\_\mathrm{g\_ya} \cdot G\_\mathrm{y}(t)$$	[32]
*Young to ancient carbonate flux:	$$F\_\mathrm{ya\_c} = k\_\mathrm{c\_ya} \cdot C\_\mathrm{y}(t)$$	[33]
Convert strontium isotopes to $^{87}Sr/^{86}Sr$:	$$\mathrm{Roc} = \frac{R\_\mathrm{ocm}}{10000} + 0.7$$	[34]
Average value of $^{87}Sr/^{86}Sr$ for young	$$R\_\mathrm{cy} = R\_\mathrm{cy}(t) + \left[(\mathrm{Roc} - R\_\mathrm{cy}(t)) \cdot \frac{F\_\mathrm{bc}}{C\_\mathrm{y}(t)}\right]$$	[35]

carbonates undergoing weathering:		
Average value of $^{87}Sr/^{86}Sr$ for ancient carbonates undergoing weathering:	$$R\_ca = R\_ca(t) + \left[(R\_cy - R\_ca(t)) \cdot \frac{F\_ya\_c}{C\_a(t)}\right]$$	[36]
Strontium fractionation of non-volcanic silicates:	$$\mathrm{Rnv} = 0.722 - \left[NV \cdot \left(\frac{1 - f\_\mathrm{RT}}{1.063}\right)\right]$$	[37]
Fraction of total Ca and Mg silicate weathering derived from volcanic rocks:	$$\mathrm{Xvolc} = \frac{\{[f\_\mathrm{SR} \cdot F\_\mathrm{bo\_0} \cdot (Rv - \mathrm{Roc})] + (F\_\mathrm{wc\_y} \cdot R\_\mathrm{cy}) + (F\_\mathrm{wc\_a} \cdot R\_\mathrm{ca}) - (\mathrm{Roc} \cdot F\_\mathrm{bc}) + (\mathrm{Rnv} \cdot F\_\mathrm{ws})\}}{F\_\mathrm{ws} \cdot (\mathrm{Rnv} - Rv)}$$	[38]
Volcanic weathering effect:	$$f\_\mathrm{volc} = \frac{(VNV \cdot \mathrm{Xvolc}) + (1 - \mathrm{Xvolc})}{(VNV \cdot \mathrm{Xvolc\_0}) + (1 - \mathrm{Xvolc\_0})}$$	[39]
*Silicate weathering:	$$F\_\mathrm{ws} = f\_\mathrm{volc} \cdot \mathrm{fBsi} \cdot (f\_\mathrm{AW} \cdot f\_\mathrm{A} \cdot f\_\mathrm{D})^{0.65} \cdot f\_\mathrm{R} \cdot f\_\mathrm{E} \cdot F\_\mathrm{ws\_0}$$	[40]

N.B. In our model code we run from -570 to 0 Ma at 10 Myr time-steps instead of +570 to 0 Ma, so some of the equations have a slightly different formulation with regard to the positive or negative sign of t or '570', compared to the original versions in GEOCARBSULF. The period -570 to -550 Ma uses the same values and formulations at -560 Ma and -570 Ma, as those used for -550 Ma, and allows for model spin-up.

* These equations have been altered from those in the original GEOCARBSULF model.

Supplementary table 6: Reservoir equations

Ocean and atmosphere sulphur:	$\frac{\mathrm{d(OA\_}S)}{\mathrm{d}t} = F\mathrm{\_wp\_y} + F\mathrm{\_wgyp\_y} + F\mathrm{\_wp\_a} + F\mathrm{\_wgyp\_a} + \mathrm{Fmp} + \mathrm{Fms} - F\mathrm{\_bp} - F\mathrm{\_bgyp}$	[41]
Ocean and atmosphere carbon (CO_2):	$\frac{\mathrm{d(CO_2)}}{\mathrm{d}t} = F\mathrm{\_wg\_y} + F\mathrm{\_wc\_y} + F\mathrm{\_wg\_a} + F\mathrm{\_wc\_a} + \mathrm{Fmg} + \mathrm{Fmca} - F\mathrm{\_bg} - F\mathrm{\_bc}$	[42]
Young pyrite:	$\frac{\mathrm{d}(Pyr\mathrm{\_y})}{\mathrm{d}t} = F\mathrm{\_bp} - F\mathrm{\_wp\_y} - F\mathrm{\_ya\_p}$	[43]
Ancient pyrite:	$\frac{\mathrm{d}(Pyr\mathrm{\_a})}{\mathrm{d}t} = F\mathrm{\_ya\_p} - F\mathrm{\_wp\_a} - \mathrm{Fmp}$	[44]
Young gypsum:	$\frac{\mathrm{d}(Gyp\mathrm{\_y})}{\mathrm{d}t} = F\mathrm{\_bgyp} - F\mathrm{\_wgyp\_y} - F\mathrm{\_ya\_gyp}$	[45]
Ancient gypsum:	$\frac{\mathrm{d}(Gyp\mathrm{\_a})}{\mathrm{d}t} = F\mathrm{\_ya\_gyp} - F\mathrm{\_wgyp\_a} - \mathrm{Fms}$	[46]
Young organic carbon:	$\frac{\mathrm{d}(G\mathrm{\_y})}{\mathrm{d}t} = F\mathrm{\_bg} - F\mathrm{\_wg\_y} - F\mathrm{\_ya\_g}$	[47]
Ancient organic carbon:	$\frac{\mathrm{d}(G\mathrm{\_a})}{\mathrm{d}t} = F\mathrm{\_ya\_g} - F\mathrm{\_wg\_a} - \mathrm{Fmg}$	[48]
Young carbonate:	$\frac{\mathrm{d}(C\mathrm{\_y})}{\mathrm{d}t} = F\mathrm{\_bc} - F\mathrm{\_wc\_y} - F\mathrm{\_ya\_c}$	[49]
Ancient carbonate:	$\frac{\mathrm{d}(C\mathrm{\_a})}{\mathrm{d}t} = F\mathrm{\_ya\_c} - F\mathrm{\_wc\_a} - \mathrm{Fmc}$	[50]
Young organic carbon isotope mass balance:	$\frac{\mathrm{d}(delta\mathrm{\_g\_y} \cdot G\mathrm{\_y})}{\mathrm{d}t} = [(delta\mathrm{\_OA\_C} - alpha\mathrm{\_C}) \cdot F\mathrm{\_bg}] - (delta\mathrm{\_g\_y} \cdot F\mathrm{\_wg\_y}) - (delta\mathrm{\_g\_y} \cdot F\mathrm{\_ya\_g})$	[51]

Ancient organic carbon isotope mass balance:	$\frac{\mathrm{d}(delta\_\mathrm{g\_a} \cdot G\_\mathrm{a})}{\mathrm{d}t} = (delta\_\mathrm{g\_y} \cdot F\_\mathrm{ya\_g}) - (delta\_\mathrm{g\_a} \cdot F\_\mathrm{wg\_a}) - (delta\_\mathrm{g\_a} \cdot \mathrm{Fmg})$	[52]
Young carbonate isotope mass balance:	$\frac{\mathrm{d}(delta\_\mathrm{c\_y} \cdot C\_\mathrm{y})}{\mathrm{d}t} = [(delta\_\mathrm{OA\_C} - alpha\_\mathrm{C}) \cdot F\_\mathrm{bc}] - (delta\_\mathrm{c\_y} \cdot F\_\mathrm{wc\_y}) - (delta\_\mathrm{c\_y} \cdot F\_\mathrm{ya\_c})$	[53]
Ancient carbonate isotope mass balance:	$\frac{\mathrm{d}(delta\_\mathrm{c\_a} \cdot C\_\mathrm{a})}{\mathrm{d}t} = (delta\_\mathrm{c\_y} \cdot F\_\mathrm{ya\_c}) - (delta\_\mathrm{c\_a} \cdot F\_\mathrm{wc\_a}) - (delta\_\mathrm{c\_a} \cdot \mathrm{Fmc})$	[54]
Atmospheric oxygen:	$\frac{\mathrm{d}(\mathrm{O_2})}{\mathrm{d}t} = \left[F\_\mathrm{bg} + \left(\frac{15}{8} \cdot F\_\mathrm{bp}\right)\right] - (F\_\mathrm{wg\_y} + F\_\mathrm{wg\_a} + \mathrm{Fmg}) - \left[\frac{15}{8} \cdot (F\_\mathrm{wp\_y} + F\_\mathrm{wp\_a} + \mathrm{Fmp})\right]$	[55]

Supplementary table 7: GEOCARBSULF sulphur isotope fractionation

Formulation used for δ^{34}S fractionation in the original GEOCARBSULF	$alpha\_S = 35 \cdot (\mathrm{O_2mr})^{1.5}$	[56]

Supplementary table 8: Equations for model tests

Fraction of the total sulphur leaving the ocean as pyrite (burial flux approach):	$f_{\mathrm{pyr}} = \frac{F\_\mathrm{bp}}{F\_\mathrm{bp} + F\_\mathrm{bgyp}}$	[57]

Fraction of the total sulphur leaving the ocean as pyrite (isotope approach)[4]:	$$f_{\mathrm{pyr}} = \frac{\delta^{34}\mathrm{S}_{\mathrm{in}} - \delta^{34}\mathrm{S}_{\mathrm{sulphate}}}{\delta^{34}\mathrm{S}_{\mathrm{sulphide}} - \delta^{34}\mathrm{S}_{\mathrm{sulphate}}}$$	[58]

Supplementary references

1. Wu, N., Farquhar, J., Strauss, H., Kim, S. T. & Canfield, D. E. Evaluating the S-isotope fractionation associated with Phanerozoic pyrite burial. *Geochim. Cosmochim. Acta* **74,** 2053–2071 (2010).

2. Sperling, E. A. *et al.* Statistical analysis of iron geochemical data suggests limited late Proterozoic oxygenation. *Nature* **523,** 451–454 (2015).

3. Wallace, M. W. *et al.* Oxygenation history of the Neoproterozoic to early Phanerozoic and the rise of land plants. *Earth Planet. Sci. Lett.* **466,** 12–19 (2017).

4. Canfield, D. E. & Farquhar, J. Animal evolution, bioturbation, and the sulfate concentration of the oceans. *Proc. Natl. Acad. Sci.* **106,** 8123–8127 (2009).

5. Halevy, I., Peters, S. E. & Fischer, W. W. Sulfate burial constraints on the phanerozoic sulfur cycle. *Science* **337,** 331–334 (2012).

6. Berner, R. A. GEOCARBSULF: A combined model for Phanerozoic atmospheric O_2 and CO_2. *Geochim. Cosmochim. Acta* **70,** 5653–5664 (2006).

7. Lenton, T. M., Daines, S. J. & Mills, B. J. W. COPSE reloaded: An improved model of biogeochemical cycling over Phanerozoic time. *Earth-Science Rev.* **178,** 1–28 (2018).

8. Bergman, N. M., Lenton, T. M. & Watson, A. J. COPSE: A new model of biogeochemical cycling over phanerozoic time. *Am. J. Sci.* **304,** 397–437 (2004).

9. Lasaga, A. C. & Ohmoto, H. The oxygen geochemical cycle:dynamics and stability. *Geochemica Cosmochim. Acta* **66,** 361–381 (2002).

10. Kanzaki, Y. & Kump, L. R. Biotic effects on oxygen consumption during weathering: Implications for the second rise of oxygen. *Geology* **45,** 611–614 (2017).

11. Berner, R. A. Addendum to 'Inclusion of the Weathering of Volcanic Rocks in the GEOCARBSULF Model': (R. A. Berner, 2006, V. 306, p. 295-302). *Am. J. Sci.* **308,** 100–103 (2008).

12. Royer, D. L., Donnadieu, Y., Park, J., Kowalczyk, J. & Goddéris, Y. Error analysis of CO_2 and O_2 estimates from the long-term geochemical model GEOCARBSULF. *Am. J. Sci.* **314,** 1259–1283 (2014).

13. Van Der Meer, D. G. *et al.* Plate tectonic controls on atmospheric CO_2 levels since the Triassic. *Proc. Natl. Acad. Sci.* **111,** 4380–4385 (2014).

14. Brune, S., Williams, S. E. & Müller, R. D. Potential links between continental rifting, CO_2 degassing and climate change through time. *Nat. Geosci.* **10,** (2017).

15. Horita, J., Zimmermann, H. & Holland, H. D. Chemical evolution of seawater during the Phanerozoic. *Geochim. Cosmochim. Acta* **66,** 3733–3756 (2002).

16. Brennan, S. T., Lowenstein, T. K. & Horita, J. Seawater chemistry and the advent of biocalcification. *Geology* **32,** 473–476 (2004).

17. Lowenstein, T. K., Timofeeff, M. N., Kovalevych, V. M. & Horita, J. The major-ion composition of Permian seawater. *Geochim. Cosmochim. Acta* **69,** 1701–1719 (2005).

18. Gill, B. C., Lyons, T. W. & Saltzman, M. R. Parallel, high-resolution carbon and sulfur isotope records of the evolving Paleozoic marine sulfur reservoir. *Palaeogeogr. Palaeoclimatol. Palaeoecol.* **256,** 156–173 (2007).

19. Algeo, T. J., Luo, G. M., Song, H. Y., Lyons, T. W. & Canfield, D. E. Reconstruction of secular variation in seawater sulfate concentrations. *Biogeosciences* **12,** 2131–2151 (2015).

20. Foster, G. L., Royer, D. L. & Lunt, D. J. Future climate forcing potentially without precedent in the last 420 million years. *Nat. Commun.* **8,** 1–8 (2017).

21. Kump, L. R. & Garrels, R. M. Modeling atmospheric O_2 in the global sedimentary redox cycle. *Am. J. Sci.* **286,** 337–360 (1986).

22. Berner, R. A. Phanerozoic atmospheric oxygen: new results using the geocarbsulf model. *Am. J. Sci.* **309,** 603–606 (2009).

23. Berner, R. A. 3GEOCARBII: A Revised Model of Atmospheric CO_2 over Phanerozoic Time. *Am. J. Sci.* **294,** 56–91 (1994).

24. Berner, R. A. & Canfield, D. E. A new model for atmospheric oxygen over Phanerozoic time. *Am. J. Sci.* **289,** 333–361 (1989).

25. Saltzman, M. R. & Thomas, E. Carbon Isotope Stratigraphy. *Geol. Time Scale 2012* **1–2,** 207–232 (2012).
